# Eukaryotic Elongation Factor 2 Kinase (eEF2K) in Cancer

**DOI:** 10.3390/cancers9120162

**Published:** 2017-11-27

**Authors:** Xuemin Wang, Jianling Xie, Christopher G. Proud

**Affiliations:** 1 South Australian Health & Medical Research Institute, North Terrace, Adelaide, SA 5000, Australia; Xuemin.Wang@sahmri.com (X.W.); Jianling.Xie@sahmri.com (J.X.); 2 Department of Biological Sciences, University of Adelaide, Adelaide, SA 5005, Australia

**Keywords:** mRNA translation, mTORC1, AMPK, eEF2, autophagy, migration, α-kinase

## Abstract

Eukaryotic elongation factor 2 kinase (eEF2K) is a highly unusual protein kinase that negatively regulates the elongation step of protein synthesis. This step uses the vast majority of the large amount of energy and amino acids required for protein synthesis. eEF2K activity is controlled by an array of regulatory inputs, including inhibition by signalling through mammalian target of rapamycin complex 1 (mTORC1). eEF2K is activated under conditions of stress, such as energy depletion or nutrient deprivation, which can arise in poorly-vascularised tumours. In many such stress conditions, eEF2K exerts cytoprotective effects. A growing body of data indicates eEF2K aids the growth of solid tumours in vivo. Since eEF2K is not essential (in mice) under ‘normal’ conditions, eEF2K may be a useful target in the treatment of solid tumours. However, some reports suggest that eEF2K may actually impair tumorigenesis in some situations. Such a dual role of eEF2K in cancer would be analogous to the situation for other pathways involved in cell metabolism, such as autophagy and mTORC1. Further studies are needed to define the role of eEF2K in different tumour types and at differing stages in tumorigenesis, and to assess its utility as a therapeutic target in oncology.

## 1. Introduction to eEF2K

Eukaryotic elongation factor 2 (eEF2) kinase (eEF2K) is an atypical protein kinase that phosphorylates and inactivates eEF2, the protein that helps ribosomes move along mRNAs during the elongation stage of protein synthesis (mRNA translation; reviewed [1,2]). Phosphorylation of eEF2 on Thr56 of its mature polypeptide renders it unable to interact with ribosomes [3] and thus inactive in translation elongation. eEF2K is therefore a negative regulator of the elongation stage of protein synthesis; this is the stage that consumes almost all (>99%) of the energy (ATP and GTP) and amino acids used by protein synthesis. The addition of each amino acid requires two GTP molecules (used directly in elongation and hydrolysed to GDP) and one ATP (used in attaching amino acids to tRNAs, which is hydrolysed to AMP, i.e., the equivalent of two high energy bonds). In many cells, protein synthesis is a major part of the cellular energy economy, using up to 30–35% of total ATP (or equivalents).

## 2. The eEF2K Protein

**Figure 1:**
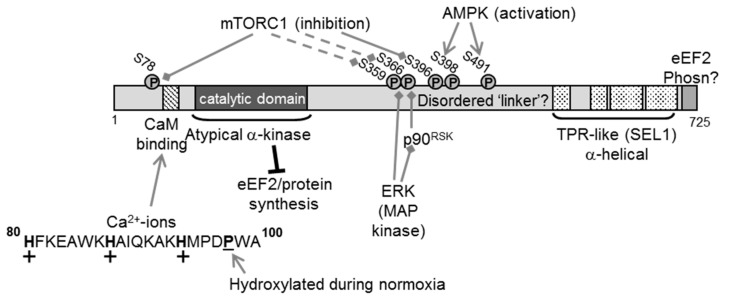
Structural layout and regulation of eEF2K. Shown are the known functional domains of the eEF2K protein. The histidines in the CaM-binding domain are shown in bold, and the ‘+’ indicates their protonation aids CaM binding under low pH conditions. The proline that undergoes hydroxylation under low oxygen conditions is underlined. Phosphorylation sites (P) and the pathways that control them are indicated (as either inhibitory or activating inputs). ERK MAP kinase signalling also regulates the sites at Ser359 (directly) and Ser366 (via their downstream kinases, p90 RSKs). ‘Phosn’, phosphorylation; ‘TPR’, tetratricopeptide; dotted lines indicate indirect signalling links; solid lines indicate direct phosphorylation events.

Human eEF2K contains 725 amino acid residues; the catalytic domain lies towards the N-terminus (Figure 1). When cDNAs for eEF2K were first cloned, it was clear that the sequence of eEF2K did not resemble those of any other known kinase [4]. As more genomic data became available, it became clear that eEF2K belongs to a small family of protein kinases, which are termed ‘α-kinases’ on the basis of their supposed preference for phosphorylating residues in α-helices (other protein kinases ‘prefer’ residues in β-turns) [5,6].

Although no crystal structure for the catalytic domain of eEF2K is available, such data are available for two other α-kinases [7,8]. The α-kinase domains of *Dictyostelium* myosin-II heavy chain kinase A (MHCK A) and the mouse transient receptor potential ion channels, TRPM7, share substantial similarities, in particular conservation of the spatial positions of their key catalytic residues. Indeed, sequence alignments and superposition on these two known 3D structures suggest strong conformation of the architecture of the kinase domain across the α-kinases. The first crystal structure of the kinase domain of an α-kinase (for TRPM7) revealed, surprisingly given the lack of sequence similarity with members of the main (‘classical’) protein kinase superfamily, that its overall architecture resembles that of other protein kinases, at least in certain respects [8]. The catalytic domains of classical and α-kinases comprise two lobes, with nucleotides being bound between them; the N-terminal lobe of the kinase domain of TRPM7 bears striking similarities to that of classical protein kinases, while the C-terminal one shows similarity to ATP-grasp proteins [8]. The latter include enzymes that catalyse the ATP-assisted reaction of a nucleophile via an acyl-phosphate intermediate [9]. Interesting, the structure of the kinase domain of MHCK A revealed just such an acyl-phosphate (acyl-aspartate [7]).

There are five further α-kinases in the human and mouse genomes, although none is as well characterised in terms of function and regulation as eEF2K. α-kinases are also found in other vertebrates (birds, amphibians, reptiles and fish), but no orthologue has been found in arthropods. Nonetheless, α-kinases are found in lower organisms such as slime moulds and nematode worms (though none is similar to eEF2K outside their catalytic domains).

Immediately N-terminal to the catalytic domain is a region that binds the Ca-sensing protein calmodulin (CaM; [10,11]) (Figure 1). Under almost all circumstances, the activity of eEF2K is dependent on Ca^2+^-ions [12,13]. The sequence of the CaM-binding motif in eEF2K does not show close similarity to the CaM-binding regions of many other proteins.

The C-terminal part of eEF2K contains four predicted SEL1-like α-helical motifs; such motifs are often involved in protein–protein interactions [14] (Figure 1). Although this C-terminal region and the extreme C-terminal end of eEF2K are required for it to phosphorylate eEF2, structural studies suggest that at least the last 99 amino acids do not provide a primary binding site for eEF2 [15]. At the extreme C-terminus is a short, highly conserved sequence that is critical for the ability of eEF2k to phosphorylate eEF2 [16].

The catalytic and SEL1 regions are connected by a region with little predicted secondary structure (that we therefore refer to as a ‘linker’) but that contains several phosphorylation sites that can regulate the activity of eEF2K.

## 3. Regulation of eEF2K

Cells suffer a range of insults, and eEF2K is regulated under a number of them, including nutrient deprivation, energy depletion, inadequate growth factor signalling and hypoxia, as well as DNA damage (reviewed in [2]).

A key initial step in the activation of eEF2K involves its autophosphorylation, which occurs on Thr348 of the human protein [17], a feature that is also known to occur in some other α-kinases including MHCK A [18]. The phosphorylated threonine is thought to slot into a binding pocket in MHCK A [18] and probably also in eEF2K [19], thereby inducing or stabilising a conformation that can phosphorylate substrates (for eEF2K, eEF2) in *trans*. It is notable that eEF2K seems to be able to undergo this activating autophosphorylation even in its initial, non-phosphorylated state, and that this can occur (slowly) even in the absence of CaM (e.g., when the protein is expressed in bacteria, which lack CaM.

On its own, the N-terminal part containing the CaM-binding site and catalytic domain cannot phosphorylate either eEF2 or a peptide substrate termed MH-1 (based on the sequence of a site for MHCK A in a myosin heavy chain [10]). When combined with a fragment containing the C-terminal SEL1 domain and when the tail is combined with the N-terminal part, activity is restored [16]. Thus the two domains work together, even in trans, to create functional enzyme (Figure 1), and can indeed form a stable complex [16].

In addition to its direct role in binding CaM, allowing eEF2K to be activated, the CaM binding motif provides two further unusual features that modulate eEF2K activity. First, it contains three histidine residues (Figure 1); histidines are the only surface residues that ionise around physiological pH, gaining protons and thus positive charge at acidic pH values (e.g., around 6.7 rather the normal pH of 7.4). eEF2K activity rises at such pH values and these histidines play a key role in this effect [20]. This is likely because the positive charge in CaM-binding domains enhances CaM binding, although the charged histidines may also take part more directly in the (unknown) events by which CaM switches on eEF2K activity.

Activation of eEF2K may be important in tissues such as muscle to slow down protein synthesis when tissue is subject to major demands for ATP and increased anaerobic glycolysis leads to acidosis, or in the tumour microenvironment [21,22].

Second, under normoxic conditions, a proline immediately C-terminal to the CaM-binding domain undergoes stoichiometric hydroxylation, which impairs the ability of CaM to activate eEF2K [23] (Figure 1). As prolyl hydroxylases require oxygen for function, this modification is impaired during hypoxia. As proline hydroxylation cannot be reversed, existing eEF2K retains the modification, but newly-made eEF2K will not be hydroxylated and will be more strongly activated by CaM. This would allow eEF2K to slow down protein synthesis and the accompanying saving of ATP, etc. under conditions where oxygen is scarce and oxidative metabolism may not be able to keep pace with cellular energy demands.

eEF2K is also extensively regulated by phosphorylation [1,2] (Figure 1). Signalling through the anabolic mammalian target of rapamycin complex 1 (mTORC1) pathway negatively regulates eEF2K activity, thereby disinhibiting eEF2. mTORC1 is a key cellular sensor of the nutrient or energy status of the cell [24]; mTORC1 function depends on the availability of essential amino acids, such as leucine and arginine, thus providing a mechanism to couple amino acid availability to control of translation elongation (and also translation initiation and ribosome production, which are also positively controlled by mTORC1 [24]).

There are several known links to eEF2K from mTORC1; for example, S6 kinase, which is activated by mTORC1, phosphorylates eEF2K on Ser366 and impairs its activation by Ca^2+^/CaM [25]. Immunoprecipitated mTORC1 directly phosphorylates Ser78/Ser396 in vitro [26], and this is inhibited by a specific inhibitor of mTOR, AZD8055. Phosphorylation of Ser78 [27] is sensitive to mTORC1 inhibition in vivo; it is less clear what contribution mTOR signalling makes to Ser396 in cells. Thirdly, several other sites are indirectly controlled by mTORC1, through mechanisms that are not well understood [26].

Oncogenic ERK (extracellular signal-regulated kinase) signalling regulates eEF2K in two main ways; (i) p90^RSK^ (p90 ribosomal protein S6 kinase), which is activated by ERK, also phosphorylates Ser366 [25] and (ii) purified ERK can directly phosphorylate Ser359 in vitro, an event that inhibits eEF2K activity [26]. Inhibition of MEK/ERK signalling impairs insulin-induced phosphorylation of this site in cells [26]. (These phosphosites are shown in Figure 1 as targets for control by mTORC1 but are also regulated by ERK signalling). Several other protein kinases are also linked to control of eEF2K, including other MAPK (mitogen-activated protein kinase) family members [28,29].

The AMP-activated protein kinase (AMPK) acts as a ‘master regulator’ linking cellular energy levels to the control of processes that use or consume metabolic energy [30]. When ATP levels fall, even slightly, there is a much greater rise in AMP (due to the action of adenylate kinase), and higher AMP concentrations cause the activation of AMPK. Agents that activate AMPK induce the phosphorylation of eEF2 [31,32,33] and inhibit protein synthesis, thus potentially saving energy. AMPK can phosphorylate eEF2K [32]; while an earlier report identified Ser398 as the target for AMPK [32], recent data show that AMPK phosphorylates eEF2K at Ser491/2 in vitro [34]. AMPK also impairs mTORC1 signalling [35,36], providing a further way in which lower cellular energy levels can turn on eEF2K.

## 4. eEF2K in Cytoprotection

As discussed below, several studies have shown that eEF2K helps to promote the survival of cancer cells in vitro, especially under nutrient-deprived conditions (reviewed [37,38,39]) or during acidosis [22]. There are also data indicating that eEF2K promotes tumour growth in vivo. In contrast, one study showed that inhibition of eEF2K actually promotes the development of intestinal cancer in a mouse model [40]. Rapidly-growing cancer cells need high rates of protein synthesis to allow the cells to proliferate. However, this creates a high demand for amino acids and metabolic energy, whereas tumours often have only a limited nutrient supply. Cells therefore need to adapt to nutrient deprivation to survive. eEF2K is activated and overexpressed in many tumours/cancers. It seems paradoxical that proliferating cells should express high levels of a negative regulator of protein synthesis, and may suggest that eEF2K has a beneficial effect in cancer cells.

**Figure 2:**
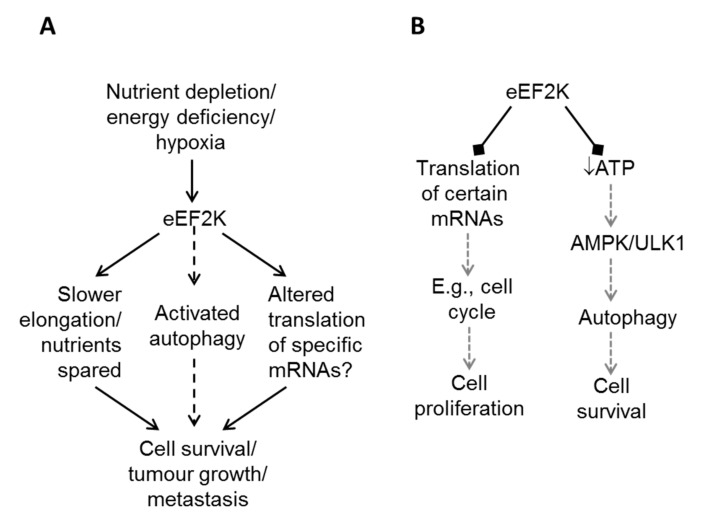
Roles of eEF2K in cancer cells. (**A**) In many types of tumour cells, and likely in more advanced solid tumours, eEF2K helps protect cells against nutrient depletion and other stresses by slowing down protein synthesis (thereby conserving resources) and/or altering the translation rates of specific mRNAs and thus the levels of the corresponding proteins. Some studies also suggest that eEF2K can promote autophagy, but this is not a general effect (dashed line). (**B**) eEF2K may also restrain tumour initiation, e.g., in colorectal cancer, by inhibiting general protein synthesis and/or the translation of specific mRNAs. Also, by maintaining ATP levels (through inhibition of protein synthesis), eEF2K may also indirectly inhibit the activation of autophagy.

Several possible mechanisms have been proposed to explain how eEF2K may promote cancer cell survival and tumour development; these are summarised in Figure 2A. First, given that protein synthesis accounts for a high proportion of cellular energy and most of their amino acid usage, eEF2K may help cells to conserve such resources, especially under conditions of nutrient starvation. Second, because regulating elongation can affect the rates of synthesis of specific proteins, activation of eEF2K might favour the synthesis of proteins important in cell survival. Third, several reports indicate that eEF2K may positively regulate autophagy (e.g., [41,42,43,44,45]), a catabolic process that, by breaking down macromolecules, may increase the available supply of amino acids, for example. Of course, additional, yet-to-be-identified mechanisms might also play a role.

An interesting study on the role of eEF2K in oocytes [46] revealed that knocking out eEF2K actually decreased the level of cell death (apoptosis) in mouse oocytes, allowing survival of poor-quality oocytes. A similar effect was seen in the nematode *C. elegans*. This is the converse of the type of effects seen in many cancer cells, where loss of eEF2K promotes cell death (generally under stressful conditions).

Hypoxia occurs in poorly-vascularised regions of solid tumours, where, together with the more limited supply of nutrients, it poses a metabolic challenge for cancer cells. eEF2K contributes to the inhibition of protein synthesis that occurs during hypoxia in MCF10A breast epithelial cells [47], and to the energy-sparing devices used by mammalian cells to cope with hypoxia [48]. eEF2K also contributes to the survival of primary neurons during hypoxia [23].

Cancer cells are typically characterized by their accelerated growth, which requires a robust increase in the rate of protein synthesis. However, even though it is a negative regulator of protein synthesis, eEF2K is activated and overexpressed in many cancers, suggesting a protective role of eEF2K in cancer cell survival.

In breast cancer cells (MCF-7 and MDA-MB-468), during nutrient deprivation or treatment with inhibitors of growth factor receptors, eEF2K is activated due to inhibition of the mTORC1/S6 kinase pathway. The authors’ data were interpreted as indicating that eEF2K positively regulates autophagy to help cells survive nutrient deprivation or insufficient growth factor signalling [39].

In neuroblastoma cells in which the oncogene MYCN is amplified, eEF2K is also activated and is required for these cells to survive nutrient withdrawal, as shown by the observation that they are very sensitive to the eEF2K inhibitor A-484954 [49]. Depletion of eEF2K (by siRNA) impairs MYCN-amplified neuroblastoma xenograft growth under conditions of caloric restriction. There is a strong correlation between p-eEF2 levels and poor prognosis in neuroblastoma [49]. However, it is not clear which mechanism accounts for the activation of eEF2K in this type of cancer.

## 5. eEF2K in Cancer Cell Survival: How Does eEF2K Promote Survival?

### 5.1. eEF2K and Cellular Nutrient Levels

Xie et al. [20] showed that silencing eEF2K caused a fall in ATP levels and phosphorylation (i.e., presumably activation) of AMPK in HT-29 and HCT116 cells, even without nutrient depletion (Figure 2B). Similarly, Xie et al. [22] and Moore et al. [23] also showed that disabling eEF2K led to a fall in ATP levels under conditions of acidosis and hypoxia, respectively. In contrast, in their very detailed studies, Leprivier et al. [50] noticed that, although transformed cells are more sensitive than non-transformed ones to nutrient deprivation, they were able to maintain ATP levels under this stress condition. Adaptation of transformed cells to withstand nutrient withdrawal involved, and required, eEF2K. Inability of cells to engage eEF2K or its upstream activator, AMPK, rendered them sensitive to a lack of nutrients. One way in which transformation might prevent activation of eEF2K is provided by the inhibitory inputs into eEF2K from the mTORC1 and ERK mitogen-activated protein (MAP) kinase pathways, which are often activated in tumour cells (e.g., cells expressing mutant (V12) Ras; Figure 1 and discussion above).

There is currently little other evidence concerning whether inhibition of elongation (achieved by activating eEF2K) does help to preserve cellular ATP levels.

### 5.2. eEF2K and the Control of the Synthesis of Specific Proteins

Early studies indicated that modulating the rate of translation elongation can exert differential effects on the synthesis of specific proteins [51]. In neurons, eEF2K can regulate the synthesis of specific proteins [52,53].

In breast cancer cells in vitro, depletion of eEF2K by RNA-mediated interference was associated with decreased levels of the ‘pro-oncogenic’ proteins c-Myc and cyclin D1 [54], and higher levels of the cell cycle inhibitory protein p27^Kip1^. It is not clear whether this reflects their altered synthesis or other effects. Bayraktar [55] showed that knockdown of eEF2K reduced the levels of cyclin D1, as well as impairing signalling pathways involved in cell migration, invasion and survival, i.e., p-Src (Tyr416), p-focal adhesion kinase (FAK) (Tyr397) and p-protein kinase B (PKB, also called AKT (Ser473)).

In glioma cells, disabling eEF2K (siRNA-mediated depletion of eEF2K or the inhibitor NH125) sensitized cells to *TNF-related apoptosis-inducing ligand* (TRAIL), i.e., increased apoptosis, and reduced expression of the anti-apoptotic protein B-cell lymphoma-extra large (Bcl-xL), but not other anti-apoptotic proteins like myeloid cell leukemia-1 (Mcl-1), X-linked inhibitor of apoptosis (XIAP) or survivin, although this effect was only apparent at the highest doses of TRAIL. They also showed that overexpression of Bcl-xL blocked TRAIL-induced death of cells depleted for eEF2K. Thus Bcl-xl plays an important role in inhibiting TRAIL-induced apoptosis in this setting, while eEF2K coordinates TRAIL-modulated Bcl-xL expression in glioma cells [56]. So a combination of TRAIL and an eEF2K inhibitor could be an effective therapy for malignant glioma.

In oocytes, levels of two rapidly-degraded anti-apoptotic proteins, cellular Fas-associating protein with death domain-like interleukin-1-converting enzyme-like inhibitory protein long form (c-FLIP_L_) and XIAP, were decreased in wild-type cells treated in such a way as to activate eEF2K (doxorubicin, an anti-cancer agent [46]). In contrast, their levels were maintained in eEF2K knockout cells. Thus, eEF2k may affect the levels of specific proteins either in a transcript-selective manner, by regulating the translation of their mRNAs, or through the global impairment of protein synthesis, which will cause greater and more rapid depletion of short-lived polypeptides. 

It has been suggested that eEF2K and eEF2 might influence the efficiency of translation of mRNAs that contain regulatory elements in their 5′-untralsated regions, such as internal ribosome entry sites (IRESs) or upstream open-reading frames (uORFs). Interestingly, the mRNAs for the pro-survival proteins XIAP [57] and Bcl-2 [58,59] each contain an IRES, so if the activity of eEF2 impairs the translation of such mRNAs, this could provide a mechanism by which eEF2K could favour survival. However, there is so far no evidence that eEF2 does affect IRES-driven translation ot translation of mRNAs that contain uORFs, and some pro-death proteins are also encoded by mRNAs that contain an IRES (e.g., Apaf-1 [60]).

### 5.3. eEF2K and Autophagy

A number of studies have reported a role for eEF2K in activating autophagy, largely using glioma cells, and also in breast cancer cell lines treated to induce endoplasmic reticulum stress [41,43,45,61,62]. This possibility ‘makes sense’ at several levels: eEF2K and autophagy are both negatively regulated by mTORC1; protein synthesis is a major user of amino acids that can be generated by autophagy; and eEF2K negatively regulates an anabolic process, so it would be logical that it positively regulates a catabolic one. Two main approaches were adopted to study the role of eEF2K, siRNA-mediated knock down or inhibition by NH125, a compound with off-target or protein-aggregating effects [63,64].

A number of the earlier studies on eEF2K and autophagy relied largely or exclusively on monitoring LC-3 (microtubule-associated protein 1A/1B-light chain 3), an upstream component of the process of autophagy, which undergoes post-translational modification (addition of phosphatidylethanolamine). This alters its electrophoretic mobility. However, LC-3 is a notoriously unreliable guide to the activation status of the autophagic machinery, as it is a component of the process, rather than purely a substrate or an end-point read-out [65]. Later studies did employ alternative readouts such as a fusion between LC3 and the green fluorescent protein, GFP (see, e.g., [41,62]). To date, no molecular mechanism has been demonstrated that would account for the proposed link between eEF2K and autophagy.

In contrast, a study using human lung carcinoma (A549) cells and embryonic fibroblasts from eEF2K knockout or control mice failed to confirm a role for eEF2K in regulating autophagy [66], although eEF2K did display the expected cytoprotective effect in the face of nutrient deprivation. The data indicated that eEF2K exerted this effect by inhibiting protein synthesis (presumably through phosphorylation of eEF2) rather than by switching on autophagy, since the effect of disabling eEF2K was counteracted by inhibiting protein synthesis with cycloheximide.

A further study [20], using colon cancer cells, indicated that, rather than activating autophagy, knockdown of eEF2K actually promoted autophagy. The authors observed that knocking down eEF2K led to a fall in cellular ATP levels and a rise in AMP, with concomitant phosphorylation (and presumably activation) of AMPK. AMPK promotes autophagy by phosphorylating the protein kinase uncoordinated-51-like kinase 1 (ULK1; reviewed [67]), and these authors’ data are consistent with the increased autophagy associated with knock down of eEF2K being dependent upon this AMPK/ULK1 link. Lastly, the effect of eEF2K knockdown was also countered by treating cells with cycloheximide, indicating that its effects are very likely due to disinhibition of eEF2 and thus translation elongation.

### 5.4. Other Roles of eEF2K in Cancer Cells

Metabolism of glucose generates pyruvate, which can then be further oxidised after entry into the Krebs cycle to generate much larger amounts of ATP than are produced by glycolysis itself. However, in many cancer cells, carbons derived from glucose are not oxidised in this way, but used to support anabolic processes. This phenomenon, whereby cancer cells rely largely on glycolysis to generate ATP, is termed the Warburg effect [68]. This metabolic switch involves a specific low-activity isoform of pyruvate kinase, PK-M2 [69], which is expressed in many tumour cells (but not generally in adult cell types). Cheng et al. [70] have reported that eEF2K promotes the expression of the M2 isoform of PK, thereby favouring Warburg metabolism. The proposed mechanism involves reduced synthesis of protein phosphatase 2A, leading to upregulation of the transcription factor Myc and hence of PK-M2. Since Myc controls the transcription of a very large number of genes, eEF2K would be positioned to exert widespread effects on gene expression, but this has not been reported.

This role of eEF2K is also surprising given that other works show PK-M2 is upregulated by signalling through mTORC1, which, as described above, is a potent negative regulator of eEF2K [71].

## 6. eEF2K in Tumour Growth

In an orthotopic model of breast cancer, siRNA-mediated knockdown of eEF2K slowed tumour growth [54]. In another murine breast cancer model [72] of triple-negative disease, mutations in the tumour suppressors phosphatase and tensin homolog (PTEN) and p53 accelerates tumorigenesis; notably, PTEN and p53 are frequently mutated in breast cancer. A screen utilising a panel of kinase inhibitors revealed that survival of cells derived from primary tumours from this model growth was impaired by either of two compounds that inhibit eEF2K (TX-1718 and NH125; please refer to comment above about this latter compound). NH125 also blocked the growth of xenografted tumours.

Caloric restriction increased cell death (apoptosis) in tumour xenografts from RasV12-transformed NIH3T3 cells [50], and eEF2K overexpression protected against this effect. Thus, eEF2K is required for the resistance of tumours to caloric restriction–induced cell death [50]. Loss of eEF2K reduced the growth of RasV12-transformed NIH3T3 xenografts under caloric restriction, again, indicating that eEF2K protects tumours against caloric-restriction induced cell death in vivo.

## 7. eEF2K Promotes the Efficacy of Other Anticancer Agents

Combination therapy with two or more anti-cancer agents is an attractive way to increase the efficacy of such agents and to help prevent the emergence of drug resistance. Disabling eEF2K, either by siRNA or using NH125, was found to sensitise glioma cells to the pro-apoptotic stimulus TRAIL [56]. As noted above, in these studies, eEF2K knockdown reduced the levels of the pro-survival protein, Bcl-xL; overexpression of this protein overcame the sensitizing effect of inhibiting or knocking down eEF2K, providing a potential mechanism for this interesting effect.

Protein kinase B, also termed AKT, promotes cell survival, and therefore its inhibition favours cell death (apoptosis). In glioma (TG098 or LN229) cells, silencing of eEF2K augmented the pro-death effects of the PKB inhibitor MK-2206 [41]. The authors’ data suggest that this is because impairing eEF2K function blunts the ability of MK-2006 to activate autophagy, a pro-survival process.

Doxorubicin (also termed Adriamycin) is used in the treatment of a number of cancers, including of the breast. In an orthotopic mouse model of breast cancer, siRNA-mediated silencing of eEF2K sensitised cells to the pro-apoptotic effect of doxorubicin [54].

Nelfinavir (NFR) was initially developed as an anti-HIV (human immunodeficiency virus) agent (it inhibits HIV aspartyl proteases); it also shows activity against tumours. Surprisingly, NFR induces the phosphorylation of eEF2K and this leads to inhibition of protein synthesis [73]. In cells that are resistant to the toxic effects of NFR, it no longer increases p-eEF2 levels. Moreover, and most relevant for this review, cells lacking eEF2K show decreased sensitivity to NFR and NFR’s ability to block tumour growth in vivo is markedly reduced when eEF2K is knocked out. While the link between NFR and control of eEF2 phosphorylation is unclear, the data clearly point to a role for activation of eEF2K (i.e., inhibition of eEF2) in this setting.

eEF2K plays a role in protecting breast cancer cells against endoplasmic reticulum (ER) stress, apparently by activating autophagy [62]. Thus, a combination of ER-stress inducer and eEF2K inhibition may have potential as a therapeutic strategy. However, eEF2K is reported to promote cell death in response to ER in (non-cancerous) fibroblasts [74], substantially complicating the picture.

## 8. eEF2K in Migration and Metastasis

Several recent studies have suggested that eEF2K aids cell migration [54,55,75,76,77,78] and may thereby play important roles in promoting cancer progression. For instance, Forkhead box M1 (FOXM1; [76]), miR-603 [55] and miR-877 [78] were found to play roles in tumorigenesis concomitantly with changes in the expression levels of eEF2K. In these studies, either suppression of eEF2K activity by its inhibitors, or genetic ablation of eEF2K, effectively slowed down cancer cell migration or invasion [55,76,77] and/or significantly prevented tumour growth in xenograft animal models [54,55,77].

The mechanism by which eEF2K promotes cancer cell migration and invasion remains to be determined; however, it was noted that, in MDA-MB-231 breast cancer cells, knocking down eEF2K reduced the phosphorylation (activation state) of focal adhesion kinase (FAK) [54], which plays a key role in cell migration and invasion. Interestingly, phosphorylation of FAK’s upstream kinase, c-Src, was also reduced. It is unclear how eEF2K influences the c-Src/FAK signalling axis.

Another recent report also showed that the eEF2K inhibitor A-484954 prevented PDGF-BB (platelet-derived growth factor subunit B homodimer)-induced vascular smooth muscle cell (VSMC) migration, although concomitantly with a drastic reduction in the rates of cell proliferation as a result of A-484954-treatment [75]. Thus, the reported effect of A-484954 on VSMC migration largely reflected inhibition of cell proliferation.

## 9. Can eEF2K Inhibition Promote Cancer?

In contrast to the great majority of the literature, Faller et al. [40] report that eEF2K actually impedes the initiation and growth of intestinal tumours, using a mouse model that mimics the effect of deletion of the adenomatous polyposis coli (*Apc*) gene. Inactivation of APC is an important factor that predisposes towards colorectal cancer and is associated with enhanced mTORC1 signalling. They show that rapamycin impairs intestinal regeneration and also intestinal tumorigenesis in the *Apc* deletion model, although the effect is reversed after discontinuation of rapamycin treatment [40]. Cells in which *Apc* had been deleted show faster protein synthesis but fewer polysomes; these features are characteristic of faster elongation rates (more rapid ‘run-off’ of ribosomes). Further, they show that eEF2K rather than other targets downstream of mTORC1 (especially eukaryotic translation initiation factor 4E-binding protein 1 (4E-BP1)) is the component that confers sensitivity to rapamycin in this model (using, for example, mice in which eEF2K has been genetically knocked out).

One important question these data raise is ‘how does eEF2K affect tumorigenesis and tumour growth’? Is it by affecting the synthesis of certain proteins? Their data show that levels of proteins involved in cell cycle control (cyclins, and the cyclin-dependent kinases CDK4 and CDK6) are elevated; in most cases, their mRNA levels were also higher (Figure 2B). However, for cyclin D3 the higher protein levels were not matched by increased mRNA, and their data imply that a loss of *Apc* accelerates elongation on the cyclin D3 mRNA, but not other mRNAs tested [40]. It is not clear why this effect on elongation rates should be specific to the cyclin D3 mRNA (what special features does it possess?), or how great the contribution of this effect to tumour initiation is.

These data are important because they indicate that inhibition of eEF2K could promote tumour initiation or growth in some cases. It remains to be clarified why this effect is observed for *Apc*-deficient colorectal cancer, whereas eEF2K promotes tumour growth in other settings.

It may well be that, as is the case for mTORC1 signalling and autophagy, eEF2K inhibition may be a ‘double-edged sword’, to use the cliché applied to the fact that agents such as rapamycin can impair tumour growth in some settings but enhance cancer proliferation in others [79]. Is this type of intestinal cancer a special case? In this regard, it is interesting to note that the study by Xie et al. [49], which concluded that eEF2K actually operated to repress autophagy, so that its silencing aided cell survival, was also performed using two distinct colon cancer cells (see also Section 5.1). Interestingly, one of these lines (HT29) expresses a truncated form of APC, while the other has wild-type *Apc*. Thus the (indirect) effect of eEF2K on autophagy is not restricted to *Apc*-deficient cells.

## 10. Inhibitors of eEF2K

eEF2K is not inhibited by staurosporine, which inhibits many of the almost 500 members of the main protein kinase superfamily, presumably because it is not a member of that family, and its active-site geometry is therefore different [80]. On one hand, this makes it harder to identify chemical entities from among existing compounds that can inhibit eEF2K; on the other, it means that compounds that do inhibit eEF2K are unlikely to affect other kinases. Several inhibitors of eEF2K have been reported (see Table in [1]).

Rottlerin [80] inhibits eEF2K but also affects various other protein kinases at lower concentrations [81]. NH125, mentioned above, was reported to inhibit eEF2K [82]. However, further detailed work revealed that it can enhance eEF2 phosphorylation in cells, probably by causing proteins to aggregate [63,64]. The compound A-484954 is reported as a specific inhibitor of eEF2K, but high micromolar concentrations are required to inhibit eEF2K within cells [64].

Two pyrido[2,3-b]pyrimidine-2,4-dione derivatives were found to inhibit eEF2K activity in vitro with submicromolar IC_50_s [83], but neither is more potent than A-484954. TX-1918 [84], originally reported as a tyrosine kinase inhibitor, also inhibits eEF2K, but its properties preclude use in vivo.

To assess the efficacy of inhibiting eEF2K as a therapy for cancer, it will be important to identify and develop better inhibitors of eEF2K, for initial validation in animal models.

## 11. Conclusions and Perspective

Despite increasing focus on the role of eEF2K in cancer, key questions remain. Although it seems clear that eEF2K generally acts to protect cancer cells against stresses such as nutrient deprivation, it is still unclear how it does this. Does it alter the translation of specific mRNAs, and thus the proteome, to tip the balance in favour of cell survival? If so, which mRNAs are these and why is their translation sensitive to changes in the activity of eEF2? Or does eEF2K simply help conserve resources (amino acids, ATP/GTP) by decreasing their consumption in protein synthesis?

Furthermore, some data suggest that the role of eEF2K in cancer may be more nuanced than simply exerting a cytoprotective effect. In particular, Faller et al. [37] report that eEF2K actually impedes tumorigenesis in intestinal cancer. Thus, eEF2K—like mTORC1 signalling and autophagy—may impact in opposing ways on tumours depending on the stage and probably the type of tumour. It is clearly crucial to know what effect eEF2K exerts at different stages in different types of cancer, and more about the underlying mechanisms.

Lastly, to learn more about the role of eEF2K in oncology—and in other disease settings—it is crucial to develop small molecule inhibitors of eEF2K that are more specific and potent than the compounds that are currently available. Given that eEF2K belongs to a small and quite distinct family of protein kinases, it should be possible to generate compounds that inhibit eEF2K without affecting members of the main kinase superfamily of around 500 enzymes.

## References

[1] Liu R., Proud C.G. (2016). Eukaryotic elongation factor 2 kinase as a drug target in cancer, and in cardiovascular and neurodegenerative diseases. Acta Pharmacol. Sin..

[2] Kenney J.W., Moore C.E., Wang X., Proud C.G. (2014). Eukaryotic elongation factor 2 kinase, an unusual enzyme with multiple roles. Adv. Biol. Regul..

[3] Carlberg U., Nilsson A., Nygard O. (1990). Functional properties of phosphorylated elongation factor 2. Eur. J. Biochem..

[4] Redpath N.T., Price N.T., Proud C.G. (1996). Cloning and expression of cDNA encoding protein synthesis elongation factor-2 kinase. J. Biol. Chem..

[5] Ryazanov A.G., Pavur K.S., Dorovkov M.V. (1999). Alpha kinases: A new class of protein kinases with a novel catalytic domain. Curr. Biol..

[6] Ryazanov A.G., Ward M.D., Mendola C.E., Pavur K.S., Dorovkov M.V., Wiedmann M., Erdjument-Bromage H., Tempst P., Parmer T.G., Prostko C.R. (1997). Identification of a new class of protein kinases represented by eukaryotic elongation factor-2 kinase. Proc. Natl. Acad. Sci. USA.

[7] Ye Q., Crawley S.W., Yang Y., Cote G.P., Jia Z. (2010). Crystal structure of the alpha-kinase domain of Dictyostelium myosin heavy chain kinase A. Sci. Signal..

[8] Yamaguchi H., Matsushita M., Nairn A.C., Kuriyan J. (2001). Crystal structure of the atypical protein kinase domain of a TRP channel with phosphotransferase activity. Mol. Cell.

[9] Fawaz M.V., Topper M.E., Firestine S.M. (2011). The ATP-grasp enzymes. Bioorg. Chem..

[10] Pavur K.S., Petrov A.N., Ryazanov A.G. (2000). Mapping the functional domains of elongation factor-2 kinase. Biochemistry.

[11] Diggle T.A., Seehra C.K., Hase S., Redpath N.T. (1999). Analysis of the domain structure of elongation factor-2 kinase by mutagenesis. FEBS Lett..

[12] Ryazanov A.G., Natapov P.G., Shestakova E.A., Severin F.F., Spirin A.S. (1988). Phosphorylation of the elongation factor 2: The fifth Ca2+/calmodulin-dependent system of protein phosphorylation. Biochimie.

[13] Nairn A.C., Palfrey H.C. (1988). Phosphorylation of elongation factor-II by Ca^2+^ calmodulin-depedent kinase-III. FASEB J..

[14] Mittl P.R., Schneider-Brachert W. (2007). Sel1-like repeat proteins in signal transduction. Cell. Signal..

[15] Will N., Piserchio A., Snyder I., Ferguson S.B., Giles D.H., Dalby K.N., Ghose R. (2016). Structure of the C-Terminal helical repeat domain of eukaryotic elongation factor 2 Kinase. Biochemistry.

[16] Pigott C.R., Mikolajek H., Moore C.E., Finn S.J., Phippen C.W., Werner J.M., Proud C.G. (2011). Insights into the regulation of eukaryotic elongation factor 2 kinase and the interplay between its domains. Biochem. J..

[17] Ruys S.P.D., Wang X., Smith E.M., Herinckx G., Hussain N., Rider M.H., Vertommen D., Proud C.G. (2012). Identification of autophosphorylation sites in eukaryotic elongation factor-2 kinase. Biochem. J..

[18] Crawley S.W., Gharaei M.S., Ye Q., Yang Y., Raveh B., London N., Schueler-Furman O., Jia Z., Cote G.P. (2011). Autophosphorylation activates Dictyostelium myosin II heavy chain kinase A by providing a ligand for an allosteric binding site in the alpha-kinase domain. J. Biol. Chem..

[19] Moore C.E., Regufe da Mota S., Mikolajek H., Proud C.G. (2014). A conserved loop in the catalytic domain of eukaryotic elongation factor 2 kinase plays a key role in its substrate specificity. Mol. Cell. Biol..

[20] Xie C.M., Liu X.Y., Sham K.W., Lai J.M., Cheng C.H. (2014). Silencing of EEF2K (eukaryotic elongation factor-2 kinase) reveals AMPK-ULK1-dependent autophagy in colon cancer cells. Autophagy.

[21] Dorovkov M.V., Pavur K.S., Petrov A.N., Ryazanov A.G. (2002). Regulation of elongation factor-2 kinase by Ph. Biochemistry.

[22] Xie J., Mikolajek H., Pigott C.R., Hooper K.J., Mellows T., Moore C.E., Mohammed H., Werner J.M., Thomas G.J., Proud C.G. (2015). Molecular mechanism for the control of eukaryotic elongation factor 2 Kinase by pH: Role in cancer cell survival. Mol. Cell. Biol..

[23] Moore C.E., Mikolajek H., Regufe da Mota S., Wang X., Kenney J.W., Werner J.M., Proud C.G. (2015). Elongation factor 2 kinase is regulated by proline hydroxylation and protects cells during hypoxia. Mol. Cell. Biol..

[24] Saxton R.A., Sabatini D.M. (2017). mTOR Signaling in growth, metabolism, and disease. Cell.

[25] Wang X., Li W., Williams M., Terada N., Alessi D.R., Proud C.G. (2001). Regulation of elongation factor 2 kinase by p90^RSK1^ and p70 S6 kinase. EMBO J..

[26] Wang X., Regufe da Mota S., Liu R., Moore C.E., Xie J., Lanucara F., Agarwala U., Dit R.S.P., Vertommen D., Rider M.H. (2014). Eukaryotic elongation factor 2 kinase activity is controlled by multiple inputs from oncogenic signaling. Mol. Cell. Biol..

[27] Browne G.J., Proud C.G. (2004). A novel mTOR-regulated phosphorylation site in elongation factor 2 kinase modulates the activity of the kinase and its binding to calmodulin. Mol. Cell Biol..

[28] Knebel A., Haydon C.E., Morrice N., Cohen P. (2002). Stress-induced regulation of eEF2 kinase by SB203580-sensitive and -insensitive pathways. Biochem. J..

[29] Knebel A., Morrice N., Cohen P. (2001). A novel method to identify protein kinase substrates: eEF2 kinase is phosphorylated and inhibited by SAPK4/p38delta. EMBO J..

[30] Hardie D.G., Ross F.A., Hawley S.A. (2012). AMPK: A nutrient and energy sensor that maintains energy homeostasis. Nat. Rev. Mol. Cell Biol..

[31] McLeod L.E., Proud C.G. (2002). ATP depletion increases phosphorylation of elongation factor eEF2 in adult cardiomyocytes independently of inhibition of mTOR signalling. FEBS Lett..

[32] Browne G.J., Finn S.G., Proud C.G. (2004). Stimulation of the AMP-activated protein kinase leads to activation of eukaryotic elongation factor 2 kinase and to its phosphorylation at a novel site, serine 398. J. Biol. Chem..

[33] Horman S., Browne G.J., Krause U., Patel J.V., Vertommen D., Bertrand L., Lavoinne A., Hue L., Proud C.G., Rider M.H. (2002). Activation of AMP-activated protein kinase leads to the phosphorylation of elongation factor 2 and an inhibition of protein synthesis. Curr. Biol..

[34] Johanns M., Ruys S.P.D., Houddane A., Vertommen D., Herinckx G., Hue L., Proud C.G., Rider M.H. (2017). Direct and indirect activation of eukaryotic elongation factor 2 kinase by AMP-activated protein kinase. Cell. Signal..

[35] Inoki K., Zhu T., Guan K.L. (2003). TSC2 mediates cellular energy response to control cell growth and survival. Cell.

[36] Gwinn D.M., Shackelford D.B., Egan D.F., Mihaylova M.M., Mery A., Vasquez D.S., Turk B.E., Shaw R.J. (2008). AMPK phosphorylation of raptor mediates a metabolic checkpoint. Mol. Cell.

[37] Leprivier G., Sorensen P.H. (2014). How does oncogene transformation render tumor cells hypersensitive to nutrient deprivation?. Bioessays.

[38] Manning B.D. (2013). Adaptation to starvation: Translating a matter of life or death. Cancer Cell.

[39] Leprivier G., Rotblat B., Khan D., Jan E., Sorensen P.H. (2015). Stress-mediated translational control in cancer cells. Biochim. Biophys. Acta.

[40] Faller W.J., Jackson T.J., Knight J.R., Ridgway R.A., Jamieson T., Karim S.A., Jones C., Radulescu S., Huels D.J., Myant K.B. (2014). mTORC1-mediated translational elongation limits intestinal tumour initiation and growth. Nature.

[41] Cheng Y., Ren X., Zhang Y., Patel R., Sharma A., Wu H., Robertson G.P., Yan L., Rubin E., Yang J.M. (2011). eEF-2 kinase dictates cross-talk between autophagy and apoptosis induced by Akt Inhibition, thereby modulating cytotoxicity of novel Akt inhibitor MK-2206. Cancer Res..

[42] Cheng Y., Li H., Ren X., Niu T., Hait W.N., Yang J. (2010). Cytoprotective effect of the elongation factor-2 kinase-mediated autophagy in breast cancer cells subjected to growth factor inhibition. PLoS ONE.

[43] Wu H., Zhu H., Liu D.X., Niu T.K., Ren X., Patel R., Hait W.N., Yang J.M. (2009). Silencing of elongation factor-2 kinase potentiates the effect of 2-deoxy-D-glucose against human glioma cells through blunting of autophagy. Cancer Res..

[44] Py B.F., Boyce M., Yuan J. (2009). A critical role of eEF-2K in mediating autophagy in response to multiple cellular stresses. Autophagy.

[45] Wu H., Yang J.M., Jin S., Zhang H., Hait W.N. (2006). Elongation factor-2 kinase regulates autophagy in human glioblastoma cells. Cancer Res..

[46] Chu H.P., Liao Y., Novak J.S., Hu Z., Merkin J.J., Shymkiv Y., Braeckman B.P., Dorovkov M.V., Nguyen A., Clifford P.M. (2014). Germline quality control: eEF2K stands guard to eliminate defective oocytes. Dev. Cell.

[47] Connolly E., Braunstein S., Formenti S., Schneider R.J. (2006). Hypoxia inhibits protein synthesis through a 4E-BP1 and elongation factor 2 kinase pathway controlled by mTOR and uncoupled in breast cancer cells. Mol. Cell. Biol..

[48] Liu L., Cash T.P., Jones R.G., Keith B., Thompson C.B., Simon M.C. (2006). Hypoxia-induced energy stress regulates mRNA translation and cell growth. Mol. Cell.

[49] Delaidelli A., Negri G.L., Jan A., Jansonius B., El-Naggar A., Lim J.K.M., Khan D., Oo H.Z., Carnie C.J., Remke M. (2017). MYCN amplified neuroblastoma requires the mRNA translation regulator eEF2 kinase to adapt to nutrient deprivation. Cell Death Differ..

[50] Leprivier G., Remke M., Rotblat B., Dubuc A., Mateo A.R., Kool M., Agnihotri S., El-Naggar A., Yu B., Prakash S.S. (2013). The eEF2 kinase confers resistance to nutrient deprivation by blocking translation elongation. Cell.

[51] Walden W.E., Thach R.E. (1986). Translational control of gene expression in a normal fibroblast. Characterization of a subclass of mRNAs with unusual kinetic properties. Biochemistry.

[52] Scheetz A.J., Nairn A.C., Constantine-Paton M. (2000). NMDA-mediated control of protein synthesis at developing synapses. Nat. Neurosci..

[53] Kenney J.W., Genheden M., Moon K.-M., Foster L.J., Proud C.G. (2015). eEF2K regulates the synthesis of microtubule-related proteins in neurons. J. Neurochem..

[54] Tekedereli I., Alpay S.N., Tavares C.D., Cobanoglu Z.E., Kaoud T.S., Sahin I., Sood A.K., Lopez-Berestein G., Dalby K.N., Ozpolat B. (2012). Targeted silencing of elongation factor 2 kinase suppresses growth and sensitizes tumors to Doxorubicin in an orthotopic model of breast cancer. PLoS ONE.

[55] Bayraktar R., Pichler M., Kanlikilicer P., Ivan C., Bayraktar E., Kahraman N., Aslan B., Ulasli M., Arslan A., Oguztuzun S. (2017). MicroRNA 603 acts as a tumor suppressor and inhibits triple-negative breast cancer tumorigenesis by targeting elongation factor 2 kinase. Oncotarget.

[56] Zhang Y., Cheng Y., Zhang L., Ren X., Huber-Keener K.J., Lee S., Yun J., Wang H.G., Yang J.M. (2011). Inhibition of eEF-2 kinase sensitizes human glioma cells to TRAIL and down-regulates Bcl-xL expression. Biochem. Biophys. Res. Commun..

[57] Holcik M., Lefebvre C., Yeh C., Chow T., Korneluk R.G. (1999). A new internal-ribosome-entry-site motif potentiates XIAP-mediated cytoprotection. Nat. Cell Biol..

[58] Warnakulasuriyarachchi D., Cerquozzi S., Cheung H.H., Holcik M. (2004). Translational induction of the inhibitor of apoptosis protein HIAP2 during endoplasmic reticulum stress attenuates cell death and is mediated via an inducible internal ribosome entry site element. J. Biol. Chem..

[59] Sherrill K.W., Byrd M.P., van Eden M.E., Lloyd R.E. (2004). BCL-2 translation is mediated via internal ribosome entry during cell stress. J. Biol. Chem..

[60] Ungureanu N.H., Cloutier M., Lewis S.M., de Silva N., Blais J.D., Bell J.C., Holcik M. (2006). Internal ribosome entry site-mediated translation of Apaf-1, but not XIAP, is regulated during UV-induced cell death. J. Biol. Chem..

[61] Ren L., Zhang J., Ma H., Sun L., Zhang X., Yu G., Guan H., Wang W., Li C. (2016). Synthesis and anti-influenza a virus activity of 6′-amino-6′-deoxy-glucoglycerolipids analogs. Mar. Drugs.

[62] Cheng Y., Ren X., Zhang Y., Shan Y., Huber-Keener K.J., Zhang L., Kimball S.R., Harvey H., Jefferson L.S., Yang J.M. (2012). Integrated regulation of autophagy and apoptosis by EEF2K controls cellular fate and modulates the efficacy of curcumin and velcade against tumor cells. Autophagy.

[63] Devkota A.K., Tavares C.D., Warthaka M., Abramczyk O., Marshall K.D., Kaoud T.S., Gorgulu K., Ozpolat B., Dalby K.N. (2012). Investigating the kinetic mechanism of inhibition of elongation factor 2 kinase by NH125: Evidence of a common in vitro artifact. Biochemistry.

[64] Chen Z., Gopalakrishnan S.M., Bui M.H., Soni N.B., Warrior U., Johnson E.F., Donnelly J.B., Glaser K.B. (2011). 1-Benzyl-3-cetyl-2-methylimidazolium iodide (NH125) induces phosphorylation of eukaryotic elongation factor-2 (eEF2): A cautionary note on the anticancer mechanism of an eEF2 kinase inhibitor. J. Biol. Chem..

[65] Klionsky D.J., Abdalla F.C., Abeliovich H., Abraham R.T., Acevedo-Arozena A., Adeli K., Agholme L., Agnello M., Agostinis P., Aguirre-Ghiso J.A. (2012). Guidelines for the use and interpretation of assays for monitoring autophagy. Autophagy.

[66] Moore C.E., Wang X., Xie J., Pickford J., Barron J., Regufe da Mota S., Versele M., Proud C.G. (2016). Elongation factor 2 kinase promotes cell survival by inhibiting protein synthesis without inducing autophagy. Cell. Signal..

[67] Dunlop E.A., Tee A.R. (2014). mTOR and autophagy: A dynamic relationship governed by nutrients and energy. Semin. Cell Dev. Biol..

[68] Warburg O., Wind F., Negelein E. (1927). The metabolism of tumors in the body. J. Gen. Physiol..

[69] Christofk H.R., Heiden M.G.V., Harris M.H., Ramanathan A., Gerszten R.E., Wei R., Fleming M.D., Schreiber S.L., Cantley L.C. (2008). The M2 splice isoform of pyruvate kinase is important for cancer metabolism and tumour growth. Nature.

[70] Cheng Y., Ren X., Yuan Y., Shan Y., Li L., Chen X., Zhang L., Takahashi Y., Yang J.W., Han B. (2016). eEF-2 kinase is a critical regulator of Warburg effect through controlling PP2A-A synthesis. Oncogene.

[71] Sun Q., Chen X., Ma J., Peng H., Wang F., Zha X., Wang Y., Jing Y., Yang H., Chen R. (2011). Mammalian target of rapamycin up-regulation of pyruvate kinase isoenzyme type M2 is critical for aerobic glycolysis and tumor growth. Proc. Natl. Acad. Sci. USA.

[72] Liu J.C., Voisin V., Wang S., Wang D.Y., Jones R.A., Datti A., Uehling D., Al-Awar R., Egan S.E., Bader G.D. (2014). Combined deletion of Pten and p53 in mammary epithelium accelerates triple-negative breast cancer with dependency on eEF2K. EMBO Mol. Med..

[73] de Gassart A., Demaria O., Panes R., Zaffalon L., Ryazanov A.G., Gilliet M., Martinon F. (2016). Pharmacological eEF2K activation promotes cell death and inhibits cancer progression. EMBO Rep..

[74] Boyce M., Py B.F., Ryazanov A.G., Long K., Minden J.S., Ma D., Yuan J. (2008). A pharmacoproteomic approach implicates eukaryotic elongation factor 2 kinase in ER stress-induced cell death. Cell Death Differ..

[75] Usui T., Nijima R., Sakatsume T., Otani K., Kameshima S., Okada M., Yamawaki H. (2015). Eukaryotic elongation factor 2 kinase controls proliferation and migration of vascular smooth muscle cells. Acta Physiol..

[76] Hamurcu Z., Ashour A., Kahraman N., Ozpolat B. (2016). FOXM1 regulates expression of eukaryotic elongation factor 2 kinase and promotes proliferation, invasion and tumorgenesis of human triple negative breast cancer cells. Oncotarget.

[77] Zhu H., Song H., Chen G., Yang X., Liu J., Ge Y., Lu J., Qin Q., Zhang C., Xu L. (2017). eEF2K promotes progression and radioresistance of esophageal squamous cell carcinoma. Radiother. Oncol..

[78] Shi Q., Xu X., Liu Q., Luo F., Shi J., He X. (2016). MicroRNA-877 acts as a tumor suppressor by directly targeting eEF2K in renal cell carcinoma. Oncol. Lett..

[79] Palm W., Park Y., Wright K., Pavlova N.N., Tuveson D.A., Thompson C.B. (2015). The utilization of extracellular proteins as nutrients is suppressed by mTORC1. Cell.

[80] Gschwendt M., Kittstein W., Marks F. (1994). Elongation factor-2 kinase: Effective inhibition by the novel protein kinase inhibitor rottlerin and relative insensitivity towards staurosporine. FEBS Lett..

[81] Davies S.P., Reddy H., Caivano M., Cohen P. (2000). Specificity and mechanism of action of some commonly used protein kinase inhibitors. Biochem. J..

[82] Arora S., Yang J.M., Kinzy T.G., Utsumi R., Okamoto T., Kitayama T., Ortiz P.A., Hait W.N. (2003). Identification and characterization of an inhibitor of eukaryotic elongation factor 2 kinase against human cancer cell lines. Cancer Res..

[83] Edupuganti R., Wang Q., Tavares C.D., Chitjian C.A., Bachman J.L., Ren P., Anslyn E.V., Dalby K.N. (2014). Synthesis and biological evaluation of pyrido[2,3-d]pyrimidine-2,4-dione derivatives as eEF-2K inhibitors. Bioorg. Med. Chem..

[84] Hori H., Nagasawa H., Ishibashi M., Uto Y., Hirata A., Saijo K., Ohkura K., Kirk K.L., Uehara Y. (2002). TX-1123: An antitumor 2-hydroxyarylidene-4-cyclopentene-1,3-dione as a protein tyrosine kinase inhibitor having low mitochondrial toxicity. Bioorg. Med. Chem..

